# Implant based differences in adverse local tissue reaction in failed total hip arthroplasties: a morphological and immunohistochemical study

**DOI:** 10.1186/1472-6890-14-39

**Published:** 2014-09-05

**Authors:** Giorgio Perino, Benjamin F Ricciardi, Seth A Jerabek, Guido Martignoni, Gabrielle Wilner, Dan Maass, Steven R Goldring, P Edward Purdue

**Affiliations:** 1 Department of Pathology, Hospital for Special Surgery, 535 East 70th Street, New York NY 10021, USA; 2 Department of Orthopedic Surgery, Hospital for Special Surgery, New York, NY, USA; 3 Department of Pathology and Diagnostics, University of Verona, Verona and Pederzoli Hospital, Peschiera, Italy; 4 Division of Research, Hospital for Special Surgery, New York, NY, USA

**Keywords:** Adverse local tissue reaction, Corrosion products, Revision arthroplasty, Synovial inflammation

## Abstract

**Background:**

Adverse local tissue reaction (ALTR) is characterized by periprosthetic soft tissue inflammation composed of a mixed inflammatory cell infiltrate, extensive soft tissue necrosis, and vascular changes. Multiple hip implant classes have been reported to result in ALTR, and clinical differences may represent variation in the soft tissue response at the cellular and tissue levels. The purpose of this study was to describe similarities and differences in periprosthetic tissue structure, organization, and cellular composition by conventional histology and immunohistochemistry in ALTR resulting from two common total hip arthroplasty (THA) implant classes.

**Methods:**

Consecutive patients presenting with ALTR from two major hip implant classes (N = 54 patients with Dual-Modular Neck implant; N = 14 patients with Metal-on-Metal implant) were identified from our prospective Osteolysis Tissue Database and Repository. Clinical characteristics including age, sex, BMI, length of implantation, and serum metal ion levels were recorded. Retrieved synovial tissue morphology was graded using light microscopy and cellular composition was assessed using immunohistochemistry.

**Results:**

Length of implantation was shorter in the DMN group versus MoM THA group (21.3 [8.4] months versus 43.6 [13.8] months respectively; p < 0.005) suggesting differences in implant performance. Morphologic examination revealed a common spectrum of neo-synovial proliferation and necrosis in both groups. Macrophages were more commonly present in diffuse sheets (Grade 3) in the MoM relative to DMN group (p = 0.016). Perivascular lymphocytes with germinal centers (Grade 4) were more common in the DMN group, which trended towards significance (p = 0.066). Qualitative differences in corrosion product morphology were seen between the two groups. Immunohistochemistry showed features of a CD4 and GATA-3 rich lymphocyte reaction in both implants, with increased ratios of perivascular T-cell relative to B-cell markers in the DMN relative to the MoM group (p = 0.032).

**Conclusion:**

Our results demonstrate that both implant classes display common features of neo-synovial proliferation and necrosis with a CD4 and GATA-3 rich inflammatory infiltrate. Qualitative differences in corrosion product appearance, macrophage morphology, and lymphocyte distributions were seen between the two implant types. Our data suggests that ALTR represents a histological spectrum with implant-based features.

## Background

Modifications in bearing surface modularity and stem designs in total hip replacement (THA) were introduced in the past two decades with the goal of reducing the incidence of aseptic loosening and instability [1,2]. One of these modifications included the metal-on-metal (MoM) bearing surface, which was combined with a metallic adapter sleeve for large heads in the early 2000s. The rationale for the revival of this bearing surface included a reduction in volumetric wear and osteolysis compared to conventional metal-on-polyethylene bearings (MoP), decreased impingement throughout range of motion, and decreased rates of dislocation [1]. A second modification to increase modularity in THA was the introduction of the dual-modular neck. This provided surgeons with increased reconstructive options to potentially match each patient’s anatomy and permit the use of a MoP or ceramic-on-polyethylene (CoP) bearing surface [2].

The unintended consequence of these implant modifications has been an increasing number of new interacting surfaces of different biomaterials, subject to short-term mechanical and no biologic testing before worldwide marketing and use [3]. This has resulted in increased MoM implant failures due to a distinct type of cellular/tissue reaction, originally reported as aseptic lymphocyte dominated vasculitis-associated lesions (ALVAL), now collectively referred in the literature as adverse local tissue reactions (ALTR) or adverse reaction to metallic debris (ARMD) [4-12]. Previous histological analyses of retrieved periprosthetic tissue have shown evidence of corrosion products, metallic debris generated by abrasion and/or surface fatigue, extensive soft tissue necrosis, combined macrophagic and lymphocytic infiltrate with variable plasmacytic and eosinophilic components, and vascular wall changes [5,13-19]. A comprehensive review describing features of periprosthetic inflammation to wear debris has been addressed in a recent review article by Gallo et al. [20]. The constellation of pathologic findings observed in response to MoM implants was encompassed under the acronym ALVAL by Willert et al. to illustrate the unique lymphocytic component and probable vascular changes not seen in other typical modes of THA failure such as osteolysis or infection [14]. Failure due to ALTR has predominantly been attributed and described for MoM bearing surfaces, but evidence of head-neck and neck-stem corrosion in modular implants has been reported to result in ALTR [4,9-12,21-23].

We hypothesized that corrosion products, as previously described in the literature, generated at different contact surfaces of THA implants could be the defining factor of ALTR irrespective of the bearing surface, and these differences would be reflected in the histologic and immunohistochemical profiles of retrieval tissue between different implant types [5,7,11]. In order to investigate this hypothesis, we compared the morphologic and immunohistochemical characteristics from retrieved periprosthetic tissue using two separate classes of implants: 1) MoM bearing surface and a metallic adapter sleeve at the head-neck taper junction and 2) conventional bearing surface (metal-on-polyethylene or metal-on-ceramic) with a dual-modular neck with tapers at the head-neck and neck-stem junctions. The purposes of this study were to analyze implant-based similarities and differences in: 1. Periprosthetic tissue structure, organization, corrosion product morphology, and cellular composition by conventional histology; and 2. Cellular composition by immunohistochemistry.

This is the first study to describe the morphologic and immunohistochemical similarities and differences between ALTR associated with different implant classes. Our results demonstrate that implant design can affect corrosion product morphology and the periprosthetic tissue pathology, and indicate that the interaction between implant design and host biology can have important clinical consequences in surveillance and outcomes of hip orthopedic implants in the future.

## Methods

### Patients

Between April 2012 and June 2013, all patients with the diagnosis of ALTR based on histological analysis were identified retrospectively from the Osteolysis Tissue Database and Repository at the Hospital for Special Surgery. This prospective database collects demographics, selected clinical data, periprosthetic tissue, and biological fluid (serum, synovial fluid) from all consenting patients undergoing revision THA with suspected ALTR. Ethical committee approval was obtained prior to this study (Institutional Review Board, Hospital for Special Surgery, Protocol Number 26085). Two groups of patients were selected, representing the two major implant classes resulting in ALTR: the Dual-Modular Neck group (DMN) had a MoP or CoP bearing surface with a dual-modular neck (cobalt-chromium-molybdenum, CoCrMo) and TMZF (titanium, molybdenum, zirconia, iron) stem (Stryker, Rejuvenate) [N = 55 hips, 54 patients], and the MoM THA group had a MoM bearing surface (CoCrMo) with a metallic adapter sleeve (CoCrMo) at the head-neck junction and titanium stem (Smith & Nephew, Birmingham THA) [N = 18 hips, 14 patients]. All polyethylene used in the DMN implant was second-generation highly cross-linked polyethylene (X3, Stryker Corporation). Exclusion criteria included previous revision arthroplasty, positive intraoperative cultures, and insufficient tissue retrieval for comparative pathologic examination (less than 5 tissue sections and more than 75% tissue necrosis at light microscopy examination on all slides examined). These two implants were selected because they are examples of recently marketed modular implants with a sufficient number of cases in our institution to allow an in-depth morphologic and immunohistochemical analysis. Preoperative serum cobalt and chromium levels were obtained by quantitative inductively coupled plasma-mass spectrometry at the operating surgeons’ discretion (ARUP Laboratories, Salt Lake City, Utah). Acetabular and stem components were recorded for each implant.

### Tissue collection and sampling

All patients suspected of having ALTR at our institution undergo magnetic resonance imaging (MRI) with multi-acquisition variable-resonance image combination (MAVRIC) scan to further reduce susceptibility artifact [24]. Findings suggestive of ALTR include bulky synovitis, extracapsular disease, tendon/intramuscular edema, and capsular avulsion [24]. Periprosthetic tissue sampling in revision cases for the implants included in the database has been standardized in our institution since September 2011, when the first cases of the two series of patients described in this report were observed. Areas of inflammation were identified preoperatively on MRI, and used as guidance for tissue sampling by the operating surgeon. Samples were taken from multiple regions around the hip joint including the periprosthetic pseudocapsule, bursal synovium, and adjacent skeletal muscle when necessary and labeled accordingly. The use of cautery was minimized to avoid compromising the tissue for histologic and molecular analysis. Additionally, acetabular and femoral bone samples, core biopsies of osteolytic areas, and/or reamings were sent separately to evaluate possible bone marrow involvement when suitable. Separate tissue samples identified by location were sent to the microbiology laboratory to rule out infection and, if sizable, retrieved after culture preparation for further histological analysis.

The project research coordinator (DM, GW) harvested biological samples with presence of the pathologist (GP) to assure consistency among all the surgeons contributing cases to the database. The tissue was retrieved fresh in labeled tissue cups from the sterile area as soon as possible and kept on ice. One pathologist (GP) performed frozen section by sampling of the fresh tissue in order to assess viability and cell composition and when feasible, a representative tissue sample was processed for RNA isolation for future investigations. Remaining tissue samples were provided between two and six sites surrounding the implant. Extensive sampling was performed at macroscopic examination with care to the orientation of the specimens, including necrotic areas and/or friable, loose material. Acetabular reaming was also collected, osteolytic areas were sampled when present, and cancellous bone was also scraped from the femoral stem and/or the acetabular shell when possible. The number of paraffin blocks containing one or two tissue sections processed per case varied from 7 to 14, to minimize sampling error due to necrosis and to ensure valuable representation of the viable tissue. Photographs of each implant and selected gross tissue specimens were taken.

### Histologic analysis

All sections were processed and embedded with standard procedures, stained routinely with hematoxylin-eosin, and examined by an experienced musculoskeletal pathologist (GP) to assess the presence of ALTR. A range of 7 – 14 sections were examined per case depending on tissue availability. Cases were scored by one investigator experienced in examining periprosthetic tissue from revision THA (GP), one experienced surgical pathologist (GM), and a third investigator trained for three months on 100 archival hip revision cases with a full spectrum of adverse reactions (BR). Investigators were blinded from clinical patient characteristics. Discrepancies in scoring were resolved by consensus agreement. The ALVAL scoring system proposed by Campbell et al., which was previously used at our institution as correlative index with MRI imaging analysis, was recorded for each case [16,25-27].

Histological sections were examined for the presence (Y) or absence (N) of synovial lining loss/hyperplasia, partial or full thickness necrosis of the neo-synovial membrane and subsynovial soft tissue, cell exfoliation, vascular wall changes, high endothelial cell venules (HEV), granulomas (sarcoidosis-like with or without central necrosis), and skeletal muscle inflammatory infiltrate (Table 1). Semi-quantitative evaluation was undertaken for grading of the macrophages [28]. Macrophages were graded on a 0–3 scale (absent, occasional, clusters, diffuse/sheets). Total lymphocytes were graded as interstitial (band-like) and/or perivascular. Perivascular lymphocytes were graded according to average lymphoctic cuff thickness using a Zeiss Axioskop 40 calibrated reticule and scored as described by Natu et al. on a 0–4 scale with absence or presence of germinal centers [17]. Neutrophils were graded on a 0–2 scale [(absent, occasional, focally numerous (>5 cells x 10 HPF)]. Plasma cells were graded on a 0–2 scale [(absent, occasional, or numerous (>10 cells per HPF)]. Eosinophils were graded on a 0–1 scale (absent or present), stromal cell cellularity was graded on a 1–3 scale (slight, moderate, marked). Results were expressed as the percentage of samples containing the selected feature.

**Table 1:** Morphologic comparison of synovial structure, cellularity, macrophage content, and bone marrow involvement between the Dual-Modular Neck and the Metal-on-Metal (MoM) total hip arthroplasty (THA) groups

**Morphologic characteristic**	**Dual-Modular Neck THA (N = 55 hips)**	**MoM THA (N = 18 hips)**	**P value**
**Synovial structure**	**Cases (%)**	**Cases (%)**	
Synovial layer loss	96.4	100.0	
Synovial layer hyperplasia	78.2	77.8	
Cell exfoliation	87.3	94.4	
Necrosis	65.5	61.1	
Vascular wall changes	18.2	16.7	
High endothelial cell venules	14.5	16.7	
Granulomas	18.2	11.1	0.482
**Cellularity**			
Macrophages			0.016*
Grade 1	16.4	5.6	
Grade 2	30.9	5.6	
Grade 3	50.9	88.9	
Lymphocytes			0.066#
Grade 1	1.8	16.7	
Grade 2	9.1	5.6	
Grade 3	52.7	61.1	
Grade 4	34.5	16.7	
Stromal Cells			0.593
Grade 1	27.3	38.9	
Grade 2	50.9	44.4	
Grade 3	16.4	11.1	
Neutrophils	10.9	11.1	
Plasma cells sparse	32.7	38.9	
Plasma cells numerous	20.0	22.2	
Eosinophils	32.7	33.3	
**Macrophage content**			
Polyethylene particles	1.8	0.0	
Metallic particles	1.8	33.3	<0.005*
Corrosion products	100	100	
Intracellular distribution	Sparse	Diffuse	
Intracellular morphology	Irregular	Globular + Irregular	
Extracellular corrosion aggregates	72.7	66.7	0.662
**Bone/bone marrow**			
Necrosis	47.1	28.6	
Macrophage infiltration	47.1	100.0	
Benign lymphocytic aggregates	35.3	28.6	
Germinal centers	17.6	0.0	

*Statistically significant at p < 0.05 for Dual-Modular Neck versus MoM THA. #trend towards statistical significance p < 0.10 for Dual-Modular Neck versus MoM THA.

Morphologic Characteristics of Synovial Tissue from Dual-Modular Neck and MoM THA Groups.

Macrophage content (polyethylene, metal, and ceramic particles) was graded according to the method used for metallic particles by Natu et al. [17] (Table 1). Presence of intracellular corrosion products was recorded and extracellular aggregates were graded on a 0–1 scale (absent or present). Presence of hemosiderin deposits and/or suture material was recorded.

Bone marrow sections were evaluated for the presence (Y) or absence (N) of necrosis of bone and marrow cellular elements, macrophage infiltration, and benign lymphocytic aggregates with or without presence of germinal centers. Results were expressed as a percentage of patients displaying each morphologic feature.

### Immunohistochemistry

Fifteen cases for each of the DMN and MoM THA groups were analyzed by immunohistochemistry. The cases from the larger DMN group were selected to be representative of the spectrum of histological patterns observed as described in the results section. Conventional immunohistochemistry was performed using standard techniques on consecutive sections (GM). Heat-induced antigen retrieval was performed using a microwave oven and 0.01 mol/L of citrate buffer. All samples were processed using a sensitive ‘Bond polymer Refine’ detection system in an automated Bond immunohistochemistry instrument (Vision-Biosystem, Menarini, Florence, Italy). Antibody dilutions and source are shown in Table 2. Commercially available monoclonal antibodies were used and each batch was tested by titration for optimal dilution on both internal and external controls. Macrophage markers were CD68 (all macrophages) and CD163 (M2 macrophages) [29,30]. The lymphocytic response was assessed by expression of CD20 for B cells and CD3, CD4, and CD8 for T cells. Expression of T-bet, GATA3, and FOXP3 was used as marker for transcription factors for Th1, Th2, and Treg cells to sub-classify the T cell distribution [31-33]. High endothelial cell venules were identified as CD123 positive cells. Mast cells were identified as CD117 positive cells [34].

**Table 2:** Description of antibodies and dilutions utilized for immunohistochemistry

**Antibody**	**Clone**	**Source**	**Dilution**
CD3	SP7	THERMO SC.	1:150
CD4	4B12	NOVOCASTRA	1:150
CD8	C8/144B	DAKO	1:200
CD20	L26	NOVOCASTRA	1:100
CD68	PG-M1	DAKO	1:50
CD123	7G3	BD Phamingen	1:100
CD163	10D6	NOVOCASTRA	1:200
GATA-3	L50-823	BD Phamingen	1:150
FOXP3	221D/D3	SEROTEC	1:200
T-bet	4B10	SANTA CRUZ	1:100
Granzyme	GrB-7	MONOSAN	1:100
CD117	T595	NOVOCASTRA	1:10

Antibody Sources and Dilutions for Immunohistochemistry.

Semiquantitative analysis was performed for evaluation of macrophage, mast cell, and HEV distributions. Evaluation of CD68 and CD163 stained sections were graded as +, ++, and +++ by three investigators (GP, GM, BR) blinded to the clinical data. CD117 staining was assessed from 0–2 [absent, occasional, numerous (>5 forms per HPF)]. Granzyme immunohistochemistry and the presence of CD123 positive HEVs were assessed by the presence (Y) or absence (N) of positive cells.

A quantitative analysis (Bioquant Osteo, Bioquant Image Analysis Corporation, Nashville, TN) was performed on all sections to evaluate lymphocytic distributions in both perivascular and interstitial regions. Two perivascular and two interstitial areas on each slide were randomly selected and evaluated at high power (×400), and lymphocytes with positive stain were counted manually by two investigators blinded to the clinical characteristics (BR, GP). The results were expressed as percentage of positive cells per mm^2^. The same areas from consecutive sections were chosen for each stain, ensuring consistency in area of evaluation. The ratios between CD20:CD3, CD4:CD8, and GATA3:T-bet on the same sections were then calculated. The CD20:CD3, CD4:CD8, and GATA3:T-bet were described as a > 2:1, 1:1, or > 1:2 ratio.

A comparison control group of periprosthetic tissue was used for immunohistochemistry. For the control group (N = 17), average age was 63.5 years (standard deviation 14.0) and 71% were females. These included three cases (N = 3) of osteoarthritis with variable amount of lymphoplasmacytic infiltrate without clinical diagnosis of rheumatic disease, three cases of periprosthetic osteolysis from polyethylene/metallic wear debris in standard THA, and three (N = 3) cases of MoM implants not examined in our series (1 resurfacing, 2 MoM THA). Average time of implantation was 30 months in these patients. Additionally, we examined all cases of preoperative native synovial tissue (time zero) available for patients in our series with ALTR and identified five cases (N = 5) with variable perivascular lymphoplasmacytic infiltrate to provide a baseline comparison. These cases underwent the same pathologic and immunohistochemical evaluation as the ALTR cases in this study. Two archival cases of pelvic lymph nodes in patients with history of total hip replacement served as negative and positive immunohistochemistry controls.

### Statistics

Categorical variables were reported as frequencies and percentages and compared between the DMN and MoM THA groups by chi-square tests. Continuous variables were summarized as means and standard deviations and compared between groups with independent samples t-tests. In cases where data was not normally distributed, a Mann–Whitney *U* test was utilized. All statistical tests were two-sided and p-values less than 0.05 were considered statistically significant. Statistical analyses were performed with SAS version 9.3 (SAS Institute, Cary, NC).

## Results

### Clinical demographics

Demographics for patients that met study criteria are summarized in Table 3. Patients were older in the DMN group (N = 55 hips in 54 patients) versus the MoM THA group (p = 0.03) (Table 3). Mean time to revision was earlier in the DMN group versus MoM THA group (21.3 [8.4] months versus 43.6 [13.8] months respectively; p < 0.005) (Table 3). Serum cobalt and chromium levels were increased in the MoM THA relative to the DMN group, however, only the chromium level in unilateral cases reached statistical significance (Table 4).

**Table 3:** Demographic data for the Dual-Modular Neck and Metal-on-Metal (MoM) total hip arthroplasty (THA) groups

**Demographic factor**	**Dual-Modular Neck THA**	**MoM THA**	**P Value**
	**N = 54 patients**	**N = 14 patients**	
	**N = 55 hips**	**N = 18 hips**	
Age (years)	67.3 (range 47 – 87)	58.6 (range 31–75)	0.03*
Female (%)	59.3	64.3	0.79
BMI (mean [SD])	27.1 (4.8)	26.6 (4.3)	0.72
Time to revision (mean [SD])	21.3 (8.4)	43.6 (13.8)	<0.005*
Campbell score (mean [SD])	8.1 (1.9)	7.4 (1.6)	0.15

SD: standard deviation. *statistically significant at p < 0.05 for Dual-Modular Neck versus MoM THA.

Demographic Factors in the Dual-Modular Neck and MoM THA Groups.

**Table 4:** Preoperative serum cobalt and serum chromium levels in patients with unilateral or bilateral arthroplasties in the Dual-Modular Neck or Metal-on-Metal (MoM) total hip arthroplasty (THA) groups

	**Dual-Modular Neck THA**	**MoM THA**	**P value**
**Serum Cobalt (ng/mL)**			
Unilateral (N = 43, N = 6)	7.5 (5.6)	29.0 (54.4)	0.581
Bilateral (N = 3, N = 5)	16.8 (1.5)	23.8 (21.2)	1.0
**Serum Chromium (ng/mL)**			
Unilateral (N = 43, N = 6)	1.2 (1.4)	11.0 (16.4)	0.004*
Bilateral (N = 3, N = 5)	2.9 (1.5)	14.6 (14.2)	0.142

Data represented as mean (standard deviation) for all values.

Preoperative Serum Ion Levels in the Dual-Modular Neck and MoM THA Groups.

*statistically significant at p < 0.05 for Dual-Modular Neck versus MoM THA.

### Histological analysis

**Figure 1:**
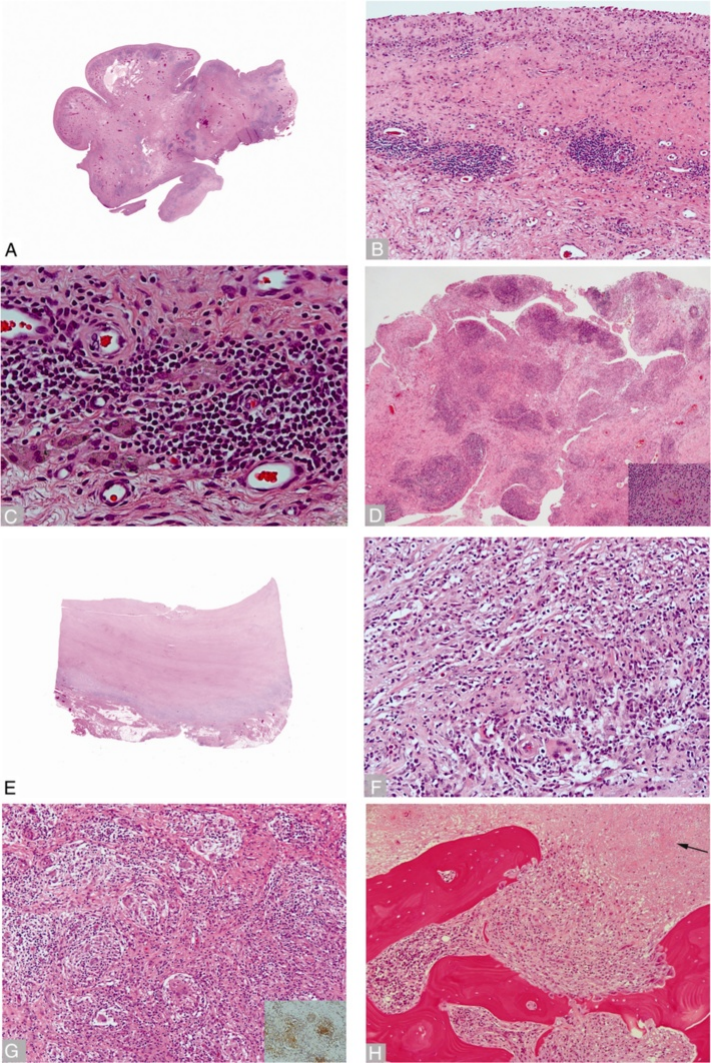
**Histopathologic images from Dual-Modular Neck group. A)** Periprosthetic neosynovium with polypoid hypertrophy (whole mount). **B)** Neo-synovial membrane with lining layer composed of macrophages, marked stromal sclerosis, and perivascular lymphocytic infiltrate (x100). **C)** Perivascular lymphocytic infiltrate associated with particle-laden macrophages (x400). **D)** Papillary synovium with marked perivascular lymphocytic infiltrate (x40) associated to giant cells containing corrosion products (inset x400). **E)** Late stage of adverse reaction with thick layer of necrosis/infarction and deep seated inflammatory infiltrate (whole mount). **F)** Band-like inflammatory infiltrate composed of macrophages, lymphocytes, plasma cells, and eosinophils (x400). **G)** Granulomatous pattern composed of small granulomas with multi-nucleated giant cells and lymphocytic cuffing (x100). **H)** Bone marrow involvement with area of necrosis (black arrow) and mixed macrophagic and lymphocytic infiltrate with resorptive osteoclastic activity (x100).

**Figure 2:**
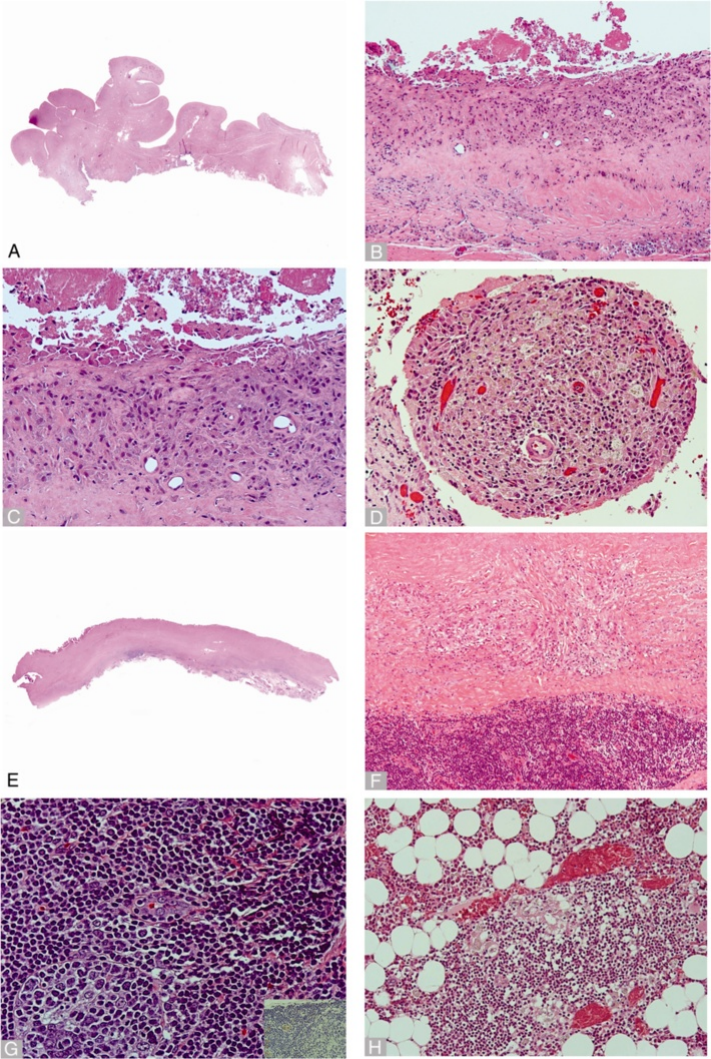
**Histopathologic images from MoM THA group A) Periprosthetic neo-synovium with polypoid hypertrophy (whole mount). B)** Superficial macrophagic infiltrate with exfoliation of necrotic elements without significant lymphocytic response (x100). **C)** Corrosion product-laden macrophages with exfoliation of viable and necrotic elements (x200). **D)** Synovial papilla with particle-laden macrophages at different stages of degeneration with foamy elements (x200). **E)** Late stage of reaction with thick layer of necrosis and deep seated inflammatory infiltrate (whole mount). **F)** Band-like mixed inflammatory infiltrate and deep perivascular lymphocytic infiltrate (x100). **G)** Evidence of germinal center formation (lower left corner) and tall endothelial cell venule (x400) with positive staining for CD123 (inset x400). **H)** Bone marrow with benign reactive lymphocytic aggregate mixed with particle-laden macrophages (x200).

Histologic examination of the retrieved tissues showed similar development of ALTR in both implants (Figures 1 and 2). Development of a reactive neo-synovial membrane ranging from flat/micropapillary, especially in bursal specimens, to florid papillary hypertrophy, ranging from coarse to polypoidal configuration, with a variable amount of stromal cell proliferation and hyperplasia of the lining layer was observed in both series (Figures 1A and 2A). The lining layer of the neo-synovium was predominantly formed by macrophages with exfoliation of viable and necrotic elements with occasional formation of a distinct eosinophilic border of coagulative necrosis, previously described in the literature as adherent fibrinous exudates (Figures 1B and 2B). An underlying band of dense sclerosis/fibroplasia and abundant macrophagic exfoliation was more commonly seen in the MoM THA (Figures 1B and 2B). Superficial perivascular lymphocytic infiltrate associated with particle-laden macrophages was commonly observed (Figures 1C and 2C). Detachment of the superficial layer or of cell contents with macrophages at different stages of degeneration (foamy elements) of the papillary projections was also observed in the MoM THA, contributing to the formation of a dense creamy fluid occasionally filling the groove of the metallic femoral head (Figure 2D). Florid papillary hypertrophy with a distribution of the inflammatory infiltrate similar to those seen in rheumatologic disorders was occasionally seen in the DMN group (Figure 1D). Tissue necrosis/infarction of variable thickness of the neo-synovial membrane, possibly reflecting more advanced stages of the reaction, was observed in 61.1% and 65.5% of cases in the MoM THA and DMN groups respectively (Table 1; Figures 1E and 2E). A deep layer of mixed macrophagic and lymphocytic infiltrate with variable number of plasma cells and eosinophils was seen in both implants (Table 1; Figures 1F and 2F). Deep perivascular lymphocytic infiltrate with formation of germinal centers and CD123 positive tall endothelial cell venules containing lymphocytes was also seen in both implants (Table 1; Figure 2G with inset). Other vascular changes were observed: the most frequent was a variable amount of non-specific myointimal proliferation of vessels with stenosis of the lumen but without identifiable luminal fibrinous exudate, thrombi, and wall damage. Occasionally onion skin pattern was present, albeit focal without evidence of a diffuse distribution in any case. Formation of sarcoidosis-like granulomas with giant cells with distinct positivity for CD123 (interleukin-3) and lymphoctic cuffing associated with the presence of corrosion products was observed in both groups (Figure 1G with inset). Higher grades of macrophagic infiltrate were seen in the MoM THA (p = 0.016) (Table 1). In contrast, higher grades of lymphocytic infiltrate were seen in the DMN group, and this trended towards significance (p = 0.066) (Table 1).

Bone marrow involvement was observed in both implants when sampled, with cell necrosis and macrophagic-lymphocytic infiltrate with associated osteoclastic activity (Figures 1H) or with benign lymphocytic aggregates associated with particle-laden macrophages (Table 1 and Figure 2H).

### Evaluation of corrosion products

**Figure 3:**
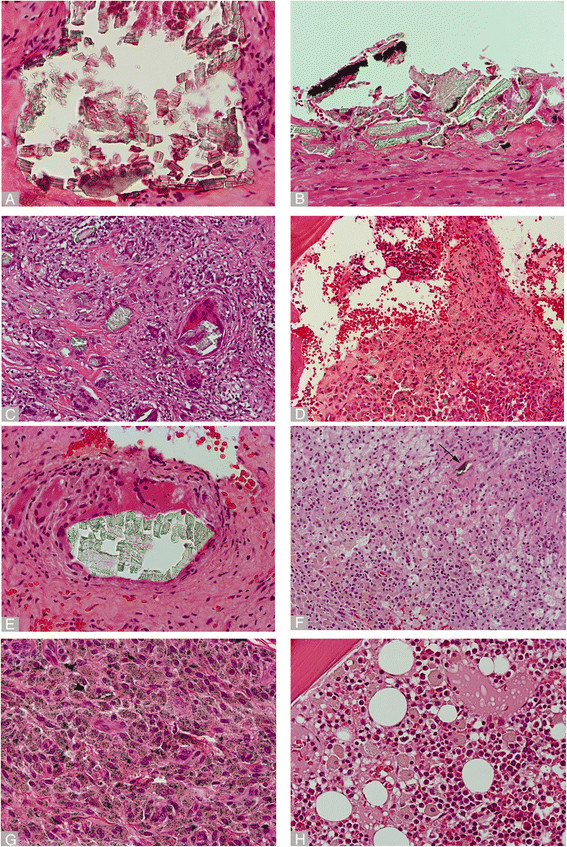
**Histopathology of corrosion products in Dual-Modular Neck and MoM THA groups.** Dual Modular Neck : **A)** Large fragments of layered corrosion products with crystal-like configuration (x400). **B)** Large aggregate of green oxidized corrosion product with plated crystal-like configuration (x400). **C)** Fragmentation of corrosion material from **(B)** engulfed into multinucleated giant cells (x200). **D)** Macrophages containing small and large particles of corrosion products infiltrating the bone marrow. MoM THA: **E)** Large aggregate of green crystal-like corrosion material surrounded by giant cells (x400). **F)** Macrophages containing corrosion products with many foamy elements and one giant cell containing larger aggregate, black arrow (x200). **G)** Macrophagic infiltrate containing predominantly globular particles of corrosion products and irregular black particles of metallic abrasion debris (x400). **H)** Hematopoietic marrow infiltrated with many macrophages containing green particles of corrosion products of variable size (x200).

Corrosion products with morphologies similar to those described in previous studies were seen in many cases in both implant types, with the exception of retrieved tissues with extensive necrosis of the superficial neo-synovial layer, in which case it was not possible to adequately assess the samples [5,11,35,36] (Figure panel 3; A-D, DMN implant; E-H. MoM THA implant). Large aggregates of corrosion products were seen in the soft tissues in both implants (66.7% versus 72.7% for MoM THA and the DMN groups respectively). A crystal-like appearance of the corrosion products with formation of flat sheets in both implant types was seen, often with alternating green and red layers on H-E staining (Figures 3A,B,E). In other areas, these larger aggregates were fragmented, and these irregular particles of variable size were engulfed within multi-nucleated giant cells and mononuclear macrophages either in soft tissue (Figure 3C) or bone marrow (Figure 3D). We confirmed the origin of these corrosion products from the implants by embedding loose black debris from the taper junctions of both implants in paraffin and obtaining H-E sections, which revealed similar morphology to those observed in the periprosthetic soft tissue of the same cases.

In contrast to these irregular extracellular aggregates, the intracellular content of the macrophages showed distinct differences between the two groups: in the DMN group the cells contained scattered irregular greenish particles which were present in large amount only in areas close to the large aggregates (Figure 3C); moreover particles were difficult to identify in the deep inflammatory layer in the cases with marked necrosis. In the MoM THA group, larger macrophages were observed, containing cytoplasmic globular particles ranging from golden brown to greenish, similar in morphology to cases of resurfacing with the same bearing surface, and scattered irregular particles. Additionally, in the MoM THA, black, irregular metallic particles were seen along with green corrosion products within the macrophages, suggesting different origins and/or compositions of metallic debris being produced in this implant type (Figure 3G). In contrast, the DMN implant did not have a metal-on-metal bearing surface, and abrasion metallic particles were not identified at light microscopy (Table 1). Bone marrow involvement of these larger aggregates and corrosion product-containing macrophages was observed in cases of both implant types if bone samples were available (Figure 3D,H).

### Immunohistochemistry

**Figure 4:**
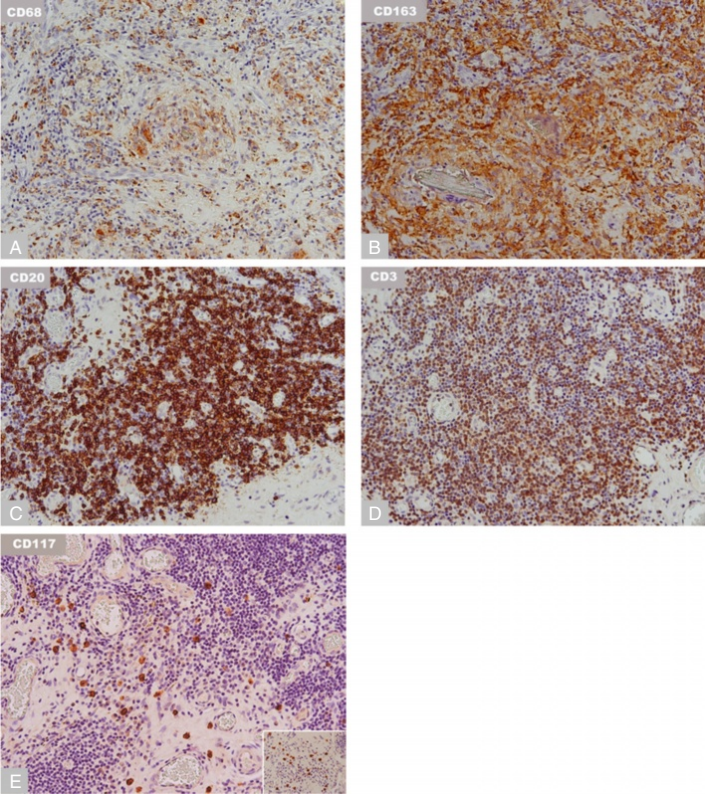
**Immunohistochemistry in the Dual-Modular Neck and MoM THA groups (all 200X magnification).** Macrophage positivity for **A)** CD68 and **B)** CD163. Perivascular lymphocytic infiltrate with **C)** CD20 positivity, **D)** CD3 positivity, and **E)** interstitial CD117 positive mast cells.

**Figure 5:**
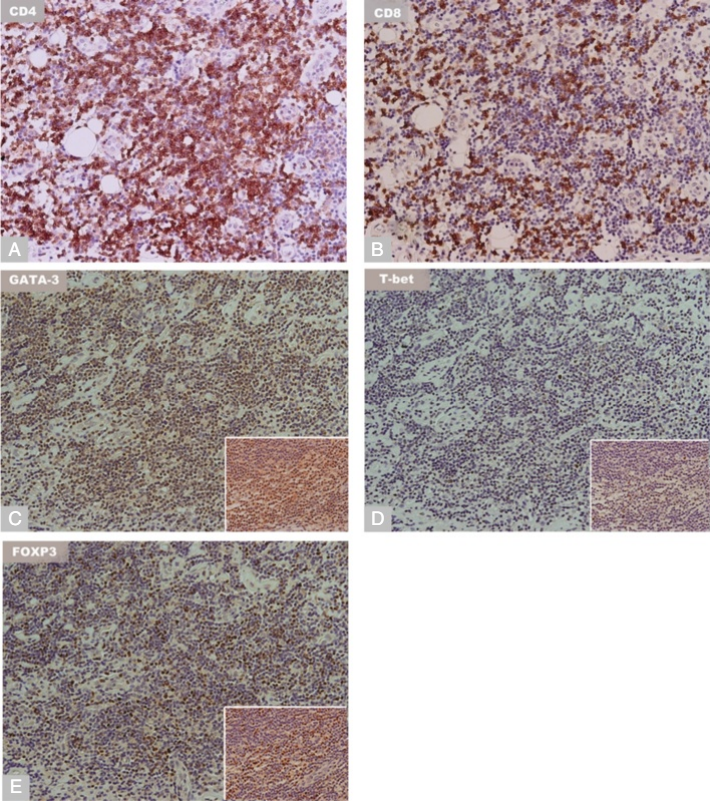
**Immunohistochemistry in the Dual-Modular Neck and MoM THA groups (all 200X magnification).** Perivascular lymphocytic infiltrate demostrates **A)** CD4 positivity and **B)** CD8 positivity. Further subclassification of perivascular lymphocytes shows **C)** T cell GATA-3 positivity, **D)** T cell T-bet positivity, and **E)** T cell FOXP3 positivity.

Both implant types had significant macrophagic infiltrates expressing CD68 and CD163 in all samples examined, with higher intensity of the latter (Figures 4A and B). Both implants displayed a mixed perivascular lymphocytic infiltrate, with increased T cell to B cell ratios in the DMN group relative to the MoM THA (Table 5; p = 0.032). In the interstitial regions, both implants had T cell predominant lymphyocytic infiltrates. The majority of patients had a mixed population of CD4 and CD8 positive T cells, with CD4 cells being more numerous in most cases in perivascular regions (Figures 5A and B; Table 5). In interstitial regions, a subset of patients in both implant types had increased CD8 positive cell density relative to CD4, however, there was no significant difference between the implant types in CD4 to CD8 ratio (p = 0.189). Further lymphocytic sub-classification showed increased number of GATA3 positive expression relative to T-bet in all samples examined (Figures 5C and D; Table 5). FOXP3 positive lymphocytes were frequently present in lymphocyte-rich regions in both implant groups, and were graded as numerous in 47% and 25% of cases in the DMN and MoM THA respectively (Figure 5E). Immunohistochemistry for granzyme showed increased presence of positive cells in the DMN group relative to the MoM THA (27% versus 0% of cases respectively), and this trended towards significance (p = 0.053). Staining for CD117 showed a variable number of mast cells, ranging from occasional to numerous (>5 cells per HPF) in interstitial and perivascular regions in both implant groups (Figure 4E).

**Table 5:** Immunhistochemistry comparison between Dual-Modular Neck and the Metal-on-Metal (MoM) total hip arthroplasty (THA) groups

**Perivascular region**				**Interstitial region**		
	**Dual-Modular Neck THA (N = 15)**	**MoM THA (N = 12)**	**P value**	**Dual-Modular Neck THA (N = 15)**	**MoM THA (N = 12)**	**P value**
CD20:CD3			*0.032			0.35
1:2	46.1%	0%		85.7%	66.7%	
1:1	30.8%	25.0%		14.3%	25.0%	
2:1	23.1%	75.0%		0%	8.3%	
CD4:CD8						
1:2	15.4%	14.3%	0.964	50.0%	16.7%	0.189
1:1	23.1%	28.6%		35.7%	66.7%	
2:1	61.5%	57.1%		14.3%	16.7%	
GATA3:Tbet						
1:2	0%	0%	1.00	0%	0%	0.42
1:1	0%	0%		38.5%	22.2%	
2:1	100%	100%		61.5%	77.8%	

All values listed as percentage of cases representing qualitative grade for each antibody. *statistically significant at p < 0.05 for Dual-Modular Neck versus MoM THA.

Immunohistochemistry Characteristics of Synovial Tissue from Dual-Modular Neck and MoM THA Groups.

Examination of control samples of retrieved revision tissues from patients with polyethylene-induced osteolysis showed no lymphocytic infiltration in any cases and a similar staining pattern for the macrophagic markers CD68 and CD163. A similar distribution of CD20:CD3, CD4:CD8, and GATA3:T-bet was seen in perivascular regions in the synovium from patients with osteoarthritis with excessive synovial chronic inflammatory infiltrate. We had the unique opportunity to evaluate native, preoperative synovial tissue from five patients in our series who subsequently developed ALTR after their total hip arthroplasty. A comparison of their native synovium with synovium after revision for ALTR showed similar lymphocytic distributions, including the presence of a CD4, GATA3 predominant perivascular lymphocytic infiltrate. Synovial necrosis was not identified in these pre-operative groups, although mild hyperplasia of the lining layer was observed. Similarly, in cases of ALTR with other MoM implants, a similar CD4 and GATA3 predominant lymphocytic infiltrate was seen.

### Histologic patterns

Three distinct histologic patterns were identified at light microscopy in this series: 1) a predominantly macrophagic pattern with absent or minimal lymphocytic response, 2) mixed inflammatory pattern, macrophagic and lymphocytic with variable presence of plasma cells, eosinophils, and mast cells, and 3) granulomatous pattern, predominant or associated with the inflammatory pattern. The predominantly macrophagic group represented a group of patients with an adverse soft tissue reaction resulting in implant failure with minimal lymphocytic activation [16,18,37]. The mixed inflammatory pattern was subdivided into those with (A) or without (B) germinal centers because this may stratify patients based on variation of the immune response [15,17,19]. The third group had prominent formation of sarcoidosis-like granulomas in the presence of a mixed or macrophagic infiltrate, and this may represent a subset of patients with particular macrophage characteristics [15,17]. In our series, a macrophagic pattern of ALTR was seen in 0% and 5.2% of cases of ALTR in the DMN and the MoM THA respectively with absent or minimal lymphocytic response (Table 6). The second, and most common, subtype was the mixed inflammatory pattern. An increased percentage of cases with germinal centers were seen in the DMN group (Table 6). Granulomatous pattern was seen more commonly in the DMN group (Table 6). In the inflammatory and granulomatous groups, ALVAL with necrosis was observed in certain cases.

**Table 6:** Observed histologic subtypes in the Dual-Modular Neck and the Metal-on-Metal (MoM) total hip arthroplasty (THA) groups

**Observed subtypes**	**Dual-Modular Neck THA (N = 54 hips)**	**MoM THA (N = 18 hips)**
**Macrophagic**	**0 (0%)**	**1 (5.2%)**
**Macrophagic with Mixed Lymphocytic Infiltrate**		
**w/ Germinal Centers**	**15 (27.2%)**	**2 (11.1%)**
**w/o Germinal Centers**	**30 (54.5%)**	**14 (77.8%)**
**Granulomatous**	**9 (16.4%)**	**1 (5.2%)**

All values listed as number (percentage of cases) representing each histologic subtype.

Histologic Subtypes of Adverse Local Tissue Reaction.

## Discussion

Failure due to ALTR has previously been described for MoM bearing surfaces and modular junctions at the head-neck and neck-stem [4,9-14,21-23,35,38,39]. The purposes of this study were to compare implant-based differences in periprosthetic tissue structure, organization, corrosion product morphology, and cellular composition by conventional histology and immunohistochemistry in ALTR resulting from two common implant configurations. Our results demonstrate that similarities between these two implants included spectrum of histologic patterns, composition of the inflammatory infiltrate, and presence of corrosion products. Differences between these implant types included macrophage and lymphocyte distributions, and corrosion product morphology. This is the first study to our knowledge to compare the histologic and immunohistochemical features of ALTR in two different classes of implants.

We have shown convincing histological evidence that similar common morphologic features exist in ALTR with an early phase of cellular activation and proliferation seen in neo-synovial reaction to other particulate implant materials (e.g. polyethylene) followed by a distinctive sequence of cellular and tissue reactions leading to formation of a variable amount of soft tissue necrosis/infarction. Corroborating evidence is provided by the metachronous development of the reaction in various areas of the periprosthetic tissue, contiguous areas of superficial necrosis, preserved neo-synovial architecture, and absence of necrosis in the bursal tissue until dehiscence of the fluid contained within the pseudocapsule. The time to revision in the DMN group was significantly shorter than the MoM THA, and this suggests different progression rates of ALTR with different implant designs. Progression of ALTR may depend on length of device implantation, toxicity/immunogenicity of corrosion particles, implant design and alignment, patient co-morbidities, and host immune reactivity. The modality of failure of the DMN and MoM THA implants analyzed in this study have been attributed in previous publications to the formation of corrosion products at the metallic interacting surfaces and not to technical mistakes or poor design resulting in mechanical failure of the implants [5,9,11]. Gill et al. also found that corrosion at the modular neck-stem junction resulted in early revision relative to the same monoblock stem and bearing components [9]. Additionally, Cooper et al. have shown a similar time to failure of the DMN implant used in our study, further corroborating our results were not due to technical error [11]. A possibility of bias in time to revision might exist because the DMN group had a publicized recall of the implant, however, all patients revised in both cohorts were indicated for revision due to elevated metal ion levels, symptomatic hip pain, MRI findings of moderate to severe adverse tissue reaction, and/or positive needle biopsies. Our observations are similar to previous studies that have illustrated the distinct histological aspects of the reaction, predominantly in MoM hip resurfacing implants or in mixed resurfacing and THA implants [10,13-19]. Previous publications examining ALTR have used the proposed ALVAL score by Campbell et al. as a grading system of the reaction [16,18,25]. If our interpretation of the natural history of the reaction is correct, the score would be an indication of developmental stage of the adverse reaction rather than a grading system of its biological severity, and therefore of limited clinical value in predicting the course or the biological outcome of the reaction for each specific type of implant.

Different histologic subtypes were observed in ALTR in our study. A subset of patients in the MoM THA group had a macrophagic pattern of failure with minimal lymphocytic response and absent or minimal necrosis. These patients may have impingement related failure, suggested by black metallic particles in their soft tissue and/or an immunoprofile that is less responsive to wear debris. A second subgroup of patients had a mixed macrophagic and lymphocytic response with a variable number of plasma cells, eosinophils, and mast cells. This has been described frequently in ALTR from previous studies and represented the most common pattern we observed [13-18]. A third subgroup displayed a granulomatous pattern with or without inflammatory infiltrate or necrosis, and this patient subgroup may have unique immunologic responses to wear debris. We did not observe any cases with an exclusively lymphocytic pattern without presence of particle-laden macrophages, as described by Berstock et al. [37]. This difference may be due to the extensive sampling performed of periprosthetic tissue in our study. The association of these different histologic patterns and clinical outcomes needs to be investigated in future studies.

We demonstrated an association between the presence of extra-cellular and intra-cellular corrosion products in the periprosthetic tissue with the presence of interstitial and perivascular lymphocytic infiltrate. This association suggests that corrosion particle laden macrophages are instrumental in the formation of the lymphocytic infiltrate, although free particulate material can also significantly contribute to the response. Corroborative evidence of our interpretation was the presence of benign lymphocytic aggregates in the bone marrow associated with particle-laden macrophages as previously reported in hip resurfacing implants [15,40]. The appearance of corrosion materials was different between the two implant designs, which also were associated with differing levels of serum cobalt and chromium ions. The MoM THA has two possible sources of corrosion materials or metallic debris: the metal-on-metal articular surface and the head-neck taper junction. The dual modular neck implant also has two possible sources of corrosion: the head-neck taper junction and the neck-stem taper junction, although the predominant one appears to be the latter [11]. These different surface possibilities likely explain the variable corrosion material appearance and distribution. Xia et al. used electron microscopy and EDX to assess macrophage content in ALVAL due to failure of a MoM bearing surface and their results showed nanometer-sized inclusions within the phagosomes with significant chromium content by EDX [41]. We hypothesize that the numerous, predominantly globular small intracellular inclusions seen on light microscopy represent corrosion products generated at the bearing surface, which are not present in the dual-modular neck implant. This observation is confirmed by the presence of the same inclusions in resurfacing implants with the same bearing surface (data not shown). In contrast to intracellular corrosion material, both implant types had large extracellular corrosion aggregates of similar morphology. Our data indicate these materials represent corrosion products from the taper junctions at the head-neck and neck-stem, which is consistent with previous studies [5,11,35]. Analysis of material produced from head-neck taper corrosion suggested that chromium orthophosphate was the most common corrosion material produced at modular junctions, and this material could disseminate into the surrounding soft tissue [5,11,35]. These wear products differ in size and shape from the intracellular products that are seen from the bearing surface, possibly explaining biological or clinical differences between different implant types [42]. Moreover, the stratified appearance of the aggregates at light microscopy possibly suggests mixing of fluid proteins and secondary particles released from the exfoliated macrophages forming products of unknown and untested cytotoxicity. Early involvement of the hematopoietic bone marrow by macrophages and large aggregates of particles can also influence the adverse reaction, and this may have future biological significance.

The ALTR reaction seen in the DMN implant is unlikely to be influenced by polyethylene debris. There have been extensive publications in the literature about wear rates of highly cross-linked polyethylene in vivo, and for the X3, femoral head penetration rates remain low at two years (head penetration <0.06 mm) [43]. Moreover, between years 1 and 5, wear rates in vivo were less than 0.001 mm/year [44]. This data suggests that polyethylene wear is unlikely to contribute to the observed reaction to ALTR seen in our study in the DMN group. This is further corroborated by the fact that only 1 of 54 cases examined in our study had polyethylene debris in their periprosthetic tissue at light microscopy, suggesting that polyethylene debris is unlikely to play a major role in ALTR seen in our study.

Immunohistochemistry results showed a predominant T lymphocytic response with a variable B cell component with the formation in some cases of perivascular germinal centers and tall endothelial cell venules as previously reported [13-17,19]. The analysis of the T cell population pointed towards a mixed pattern with predominant GATA3 positivity (Th2 lymphocytes) but also substantial T-bet and FOXP3 expressing lymphocytes, representing Th1 and Treg subgroups respectively. These findings were associated with the presence of a population of macrophages strongly positive for CD163, a marker of M2 macrophages, a subset frequently correlated with Th2 cytokines [45]. The frequent finding of a variable number of CD117 positive mast cells is also a new important finding with implications in reaction initiation/progression due to their interactions between T and B cell lymphocytes and eosinophils, and their potential to produce M2 inducing cytokines such as IL-4 [46]. Reaction initiation and severity may be explained by the release of chemokines from macrophages under oxidative stress and/or direct lymphocyte cytotoxicity [47-50]. Similar lymphocyte distributions were observed in the cases of osteoarthritis at time zero, and the possibility of a non-specific common pathway in different inflammatory conditions of the synovial membrane not representative of the initial response of the adverse reaction must be considered and confirmatory studies with testing of other specific antibodies are needed. It is also possible that the lymphocyte distributions seen in our study reflect an innate immunologic profile of the synovium with subsequent adaptive modulation, and analysis of pathologic gene expression patterns could be helpful to elucidate the role of these lymphocytic sub-populations in initiation and progression of ALTR [51]. Collectively, the immunohistochemistry studies indicate a complex adaptive immune response potentially involving several cell types. Future molecular analysis will help define the signaling pathways that orchestrate the tissue necrosis and other pathologies underlying ALTR.

The main limitation of this study is the attempt to reconstruct the natural history of the reaction based on one cross sectional observation at the time of implant revision. We compensated for this limitation by extensive topographical sampling of the periprosthetic soft tissue, but we acknowledge that continued longitudinal observation would be needed to confirm our findings.

## Conclusion

In conclusion, a common spectrum of neo-synovial proliferation and subsequent necrosis are observed in both implant classes. These findings can represent temporal progression of the reaction, which could have implant-based and patient-based characteristics. The Campbell-ALVAL score would represent an index of the staging of this temporal progression and not of the grading of the severity of ALTR. Cellular composition showed subtle differences in macrophagic and lymphocytic distributions in the two implant classes, suggesting biological differences may exist between different implant classes. The prominence of corrosion products is a consistent feature of ALTR in both implant types; however, their morphologies differ based on implant design. Immunohistochemistry showed a complex adaptive immune response, and future studies on molecular signaling pathways in ALTR are needed. The immunogenicity and toxicity of the new particulate material formed at the implant interacting surfaces and their association with hematopoietic marrow cells are still unknown, especially in patients with pre-existing immunologic disease. Short- and long-term follow-up of all patients affected by ALTR is needed to monitor for local and systemic effects.

## Abbreviations

ALTR: Adverse local tissue reaction; DMN: Dual-modular neck implant; MoM: Metal-on-metal implant; THA: Total hip arthroplasty; ALVAL: Aseptic lymphocyte dominated vasculitis-associated lesions.

## Competing interests

There are no competing financial or non-financial interests in direct relation to this manuscript for any authors. Author S Jerabek is a consultant for Mako Surgical Corporation.

## Authors’ contributions

GP conceived of the study, collected and analyzed synovial tissue, and drafted the manuscript. BR assisted with histologic and immunohistochemistry analysis and drafted the manuscript. SAJ analyzed the clinical data. GM performed immunohistochemistry and assisted with study design. GW and DM collected synovial tissue and assisted with manuscript preparation. SRG participated in study design and interpretation of data. PEP participated in study design and coordination. All authors read and approved the final manuscript.

## Pre-publication history

The pre-publication history for this paper can be accessed here:

http://www.biomedcentral.com/1472-6890/14/39/prepub
